# A Scoping Review: Urinary Markers of Metabolic Maturation in Preterm Infants and Future Interventions to Improve Growth

**DOI:** 10.3390/nu14193957

**Published:** 2022-09-23

**Authors:** Luise V. Marino, Simone Paulson, James J. Ashton, Charlotte Weeks, Aneurin Young, John V. Pappachan, Jonathan Swann, Mark J. Johnson, Robert Mark Beattie

**Affiliations:** 1 Paediatric Intensive Care Unit, Southampton Children’s Hospital, NIHR Southampton Biomedical Research Centre, University Hospital Southampton, NHS Foundation Trust, Southampton S016 6YD, UK; 2 Faculty of Health Science, University of Southampton, Southampton SO17 1BJ, UK; 3 NIHR Southampton Biomedical Research Centre, University Hospital Southampton, NHS Foundation, Southampton S016 6YD, UK; 4 Paediatric Gastroenterology, Southampton Children’s Hospital, NIHR Southampton Biomedical Research Centre, University Hospital Southampton, NHS Foundation Trust, Southampton S016 6YD, UK; 5 Human Genetics and Genomic Medicine, University of Southampton, Southampton SO17 1BJ, UK; 6 Department of Neonatal Medicine, Southampton Children’s Hospital, University Hospital Southampton, NHS Foundation Trust, Southampton S016 6YD, UK; 7 Faculty of Medicine, University of Southampton, Southampton SO17 1BJ, UK; 8 Biomolecular Medicine, School of Human Development and Health, Faculty of Medicine, University of Southampton, Southampton SO17 1BJ, UK

**Keywords:** infants, growth, preterm, urinary metabolites, metabolic maturation

## Abstract

**Background:** Growth failure in infants born preterm is a significant issue, increasing the risk of poorer neurodevelopmental outcomes and metabolic syndrome later in life. During the first 1000 days of life biological systems mature rapidly involving developmental programming, cellular senescence, and metabolic maturation, regulating normal growth and development. However, little is known about metabolic maturation in infants born preterm and the relationship with growth. **Objective:** To examine the available evidence on urinary markers of metabolic maturation and their relationship with growth in infants born preterm. **Eligibility criteria:** Studies including in this scoping review using qualitative or quantitative methods to describe urinary markers of metabolic maturation and the relationship with growth in infants born preterm. **Results:** After a screening process 15 titles were included in this review, from 1998–2021 drawing from China (*n* = 1), Italy (*n* = 3), Germany (*n* = 3), Greece (*n* = 1), Japan (*n* = 2), Norway (*n* = 1), Portugal (*n* = 1), Spain (*n* = 2) and USA (*n* = 1). The included studies examined urinary metabolites in 1131 infants. A content analysis identified 4 overarching themes relating to; (i) metabolic maturation relative to gestational age, (ii) metabolic signature and changes in urinary metabolites over time, (iii) nutrition and (iv) growth. **Conclusion:** The results of this scoping review suggest there are considerable gaps in our knowledge relating to factors associated with metabolic instability, what constitutes normal maturation of preterm infants, and how the development of reference phenome age z scores for metabolites of interest could improve nutritional and growth outcomes.

## 1. Introduction

Globally, an estimated 15 million infants are born preterm (before 37 weeks gestational age) each year, with a prevalence of 5 to 18% depending on country of birth [1]. Current recommendations suggest the growth of preterm infants should aim to approximate the in utero growth of infants of the equivalent gestation [2,3,4], although defining optimal growth relative to short and long term outcomes continues to be debated [5]. During the first 1000 days growth not only involves increasing weight and body length, but also rapid maturation of the immune system, endocrine system and metabolic pathways [6,7,8]. Post-natal growth failure in preterm infants is a persistent problem and may result in poorer neurocognitive outcomes [5], as well as increasing the risk of morbidity and mortality [5,9,10]. Conversely rapid weight gain, particularly between 2.5 and 6 years of age, is associated with the development of metabolic syndrome and cardiovascular disease later in life [11,12]. Reasons for constrained growth are numerous but may include (i) failure to deliver sufficient nutrition, (ii) intestinal immaturity resulting in alterations of nutrient utilisation by the intestine or losses via renal system, (iii) metabolic immaturity leading to transient intolerance of lipids and glucose, (iv) dysregulated maturation of metabolic pathways and urinary losses of important metabolites, (v) dysbiosis of the microbiome with poor diversity and low abundance of intestinal microbiota, (vi) medical management including use of pharmacopeia (i.e., diuretics) increasing urinary losses of electrolytes and (vii) disruption in achieving nutrition targets [5,6,13,14,15].

Although there are well established nutritional recommendations from various expert groups pertaining to macro- and micronutrient requirements of preterm infants [4,16], the recommendations do not account for an individual preterm infant’s ability to assimilate nutrients or the ability to overcome potential aberrance in metabolic pathways [5,9,17]. As an example, some preterm infants, (especially those around the threshold of viability) experience metabolic immaturity and instability leading to sustained periods of hyperglycaemia and hypertriglyceridemia, meaning that some nutritional goals are not met during a time of rapid growth and organ development [9,17]. Current strategies to manage metabolic complications include the use of insulin (which is not without risk), or potentially an even cruder strategy of reducing the amount substrate (e.g., glucose and lipid) delivered with associated negative sequalae on macro- and micronutrient intake [9,10,17,18].

With the advent of high throughput analytic techniques to quantify components of biological samples, it is increasingly possible to consider the development a more nuanced approach to medical and nutritional management for a whole range of conditions [19]. To this end ^1^H nuclear magnetic resonance (^1^H-NMR) spectroscopy and mass spectroscopy (MS) can be used to analyse the metabolome in biological fluids such as urine [20,21] and identify signatures associated with different health and disease states [13]. Although blood has been comprehensively studied with regard to metabolomic analysis, preterm infants have small circulating blood volumes. As urine is chemically complex, metabolomic analysis has been shown to provide information on varying physiological states, metabolism signatures and functions [22]. In addition, urine is readily available, collection is non-invasive and easy making it an accessible biological fluid to study. Giallourou et al. used urinary metabolic profiling to study metabolic maturation of infants (*n* = 1131) from resource constrained settings over 3 continents in the first 1000 day. From this work they identified eight metabolic signatures which were independent of feeding practices. These were developed into time dependent variation in healthy compared to growth constrained infants phenome age for z scores (PAZ) [13]. In this setting the development of PAZ for the metabolites of interest provided the opportunity to plot individual metabolic maturity in real time and provide the opportunity to offer interventions targeted to an infant’s precise metabolic predisposition [13]. Developing PAZ scores for preterm infants may provide a better understanding of metabolic factors which may be contributing to extra uterine growth retardation [23].

Developing a better understand of dynamic changes to post-natal metabolic stability and maturity in preterm infants, may help to (i) develop normative z-scores for age for metabolites associated with metabolic stability and maturation in preterm infants, (ii) refine our understanding of nutritional needs based on metabolic maturity rather than chronological maturation, (iii) provide an opportunity to identify potential future targets for nutritional supplementation to promote metabolic maturation and improve growth outcomes [13,24,25,26,27].

A scoping review was chosen over a systematic review as the use of urinary metabolomics to quantify metabolic stability and maturity in preterm infants is a relatively unexplored area of nutritional and metabolic research. As a result, it was not possible to complete a systematic review with/without meta-analysis. The rationale for this methodological approach is explored by Munn et al. [28] further. This scoping review was carried out to gain a better understanding of where the current evidence base is in terms of achieving these goals.

## 2. Materials and Methods

We chose to complete a scoping review, as a method to systematically review the available literature completing a content and narrative review.

### 2.1. Preparing to Scope the Literature and Protocol Development

A scoping review was conducted to understand the range of evidence currently available and to map key concepts within it. Specifically, it aimed to address the question “Are there specific metabolic signatures which could be used to develop reference phenome age z score for metabolites of interest associated with metabolic maturation and growth?” For the purposes of this review, we defined preterm infants as born <37 weeks gestational age.

Scoping review methodology was chosen because it offers a framework to examine a broad range of evidence in an emerging field [28] and allows the analysis of current knowledge gaps and future research priorities. The Preferred Reporting Items for Systematic reviews and Meta-Analyses extension for Scoping Reviews (PRISMA-ScR) [29] was used to report the evidence examined in this review.

### 2.2. Protocol Development

The protocol was developed using the PRISMA-ScR checklist [29] and previously published work [30]. The protocol described; (1) the research question, (2) the information sources to be searched, (3) a description of the full electronic search strategy, (4) study inclusion and exclusion criteria (5) data extraction and charting, (6) collation of data, analysis, and critical appraisal to answer the research questions posed.

### 2.3. Data Sources Searched

The research questions were used to complete a literature search across multiple databases and thus identify relevant studies. The databases searched were PubMed, the Cochrane Library, NHS Evidence and the NICE Healthcare Databases Advanced Search website (HDAS) (https://hdas.nice.org.uk/) (accessed 30 January 2022). HDAS was used to allow searches within multiple databases, including AMED, BNI, Cinahl, Embase, Health Business Elite, HMIC, Medline and PsycInfo.

### 2.4. The Search Strategy

A search strategy was devised with the assistance of a PubMed information specialist. The search strategy used key words from articles relating to infants born preterm (Appendix Table A1 and Table A2). Searches were adapted for the additional electronic databases. Forward and backward citation searching was completed on full text articles selected with no predefined start date until February 2022.

### 2.5. Study Selection

Studies were eligible for inclusion if they were written in the English language, describing urinary markers of metabolic maturation or growth in preterm infants. Opinion pieces, editorials and congress abstracts were excluded as per the scoping methodology advocated by Aksey and O’Malley [31]. Article titles and abstracts were screened, duplicates deleted, and then full text articles reviewed for eligibility (SP, LVM, JJA, CW). Where multiple articles described the same cohort of children these were only counted once. Bibliographies of included studies were hand searched for additional studies which may fulfil the inclusion criteria. Exclusion criteria included studies, infants with other primary pathologies and metabolites described in other fluids. 

### 2.6. Data Extraction and Charting

Data extraction was completed using a two-stage process. For quality control article titles and abstracts were screened, duplicates deleted, and then full text articles reviewed for eligibility (SP, LVM, JJA, CW). A data extraction template (Microsoft 2010, Redmond, WA, USA) was used to capture the study design, results, and conclusions. This was followed by content analysis.

### 2.7. Collating, Summarising and Reporting Results

Data synthesis was completed using a content analysis approach. Content analysis was chosen as it is an established technique for reporting subjects common to multiple data sets [31,32]. Descriptive aspects about the population studied, methodology, outcomes and any key findings were coded. Content analysis was completed by coding initial themes, which were grouped into sub-categories and then into overarching themes. The overarching themes and sub-categories from this process were used to develop a summary table. A narrative data synthesis was also completed [33] summarising results of the identified studies.

## 3. Results

### 3.1. Study Characteristics

**Figure 1:**
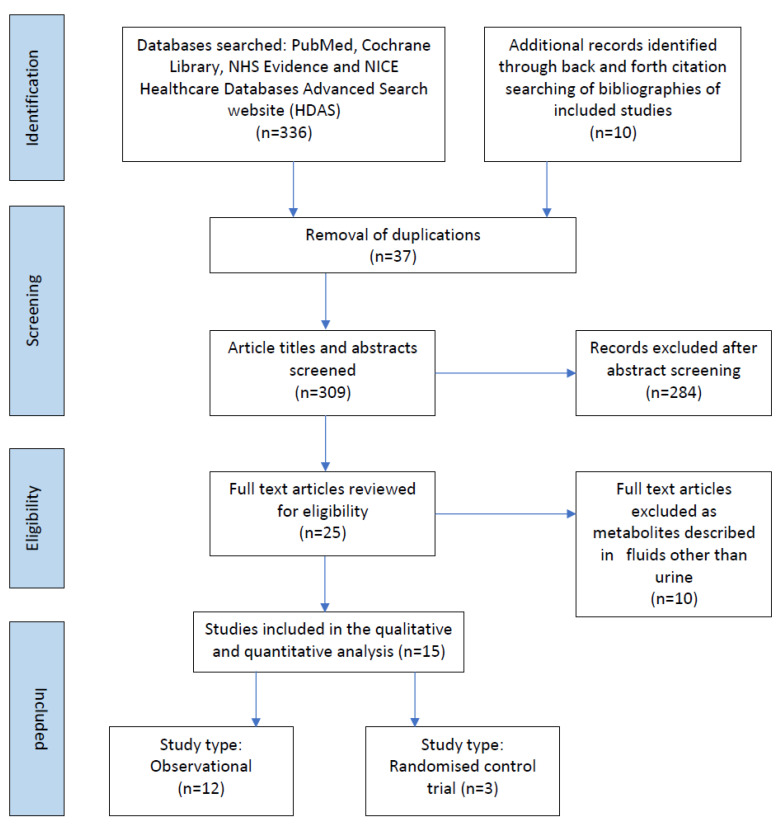
Prisma flow chart of studies included in the scoping review.

336 records were identified, of which 37 were duplicates. Following the removal of duplicate records, 309 records abstracts and titles were screened for inclusion (Figure 1). The full texts of 25 articles were reviewed for eligibility, of which 15 related to preterm infants, from 1998–2021 drawing from China (*n* = 1), Italy (*n* = 3), Germany (*n* = 3), Greece (*n* = 1), Japan (*n* = 2), Norway (*n* = 1), Portugal (*n* = 1), Spain (*n* = 2) and USA (*n* = 1). The included studies examined urinary metabolites in 1131 infants.

### 3.2. Narrative Data Synthesis

A narrative data synthesis identified preterm birth was associated with deficiencies in amino acid, carbohydrate, and fatty acid metabolism pathways and metabolites associated with energy and protein pathways are downregulated (Table 1) [34,35,36,37,38,39,40,41,42,43,44,45,46].

### 3.3. Content Analysis and Overarching Themes

**Figure 2:**
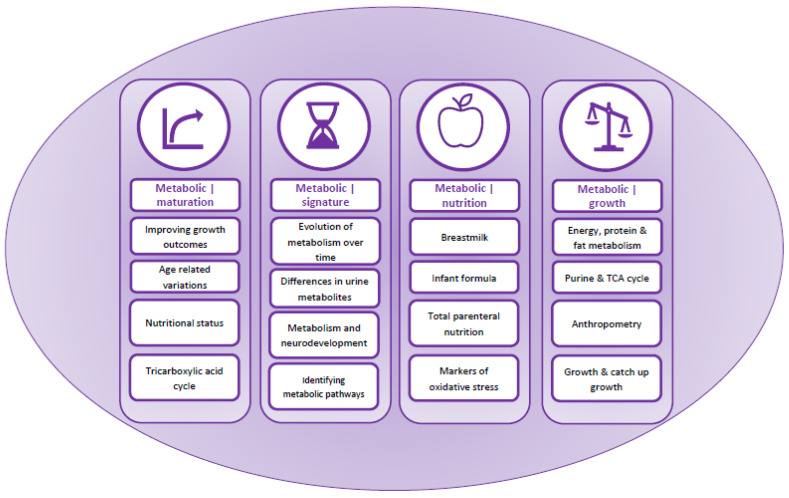
Graphical representation of the narrative synthesis and content analysis.

**Figure 3:**
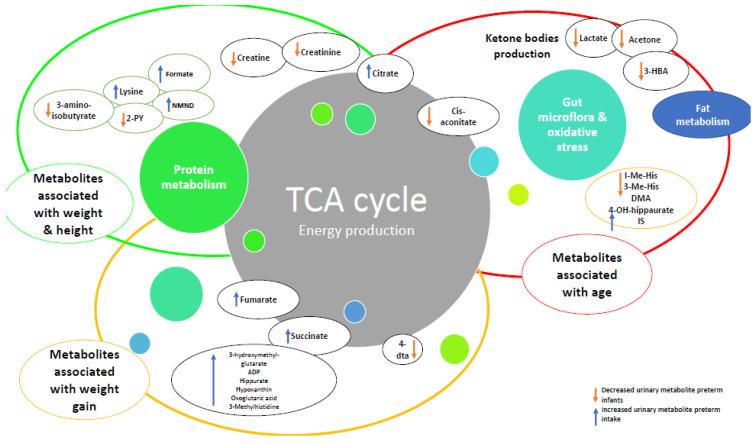
Relationships with changes in metabolites over time and growth in preterm infants compared to healthy term infants. Abbreviations: ADP: adenosine triphosphate, 4-DTA: 4-deoxythreonic acid, 4-DTA: 4-deoxythreonic acid, 3-HBA: 3-hydroxybutyrate; IS: indoxyl sulfate; 3-Me-His: 3-methylhistidine; 1-Me-His: 1-methylhistidine.

Content analysis identified four overarching themes relating to, (i) metabolic maturation relative to gestational age, (ii) metabolic signature and changes in urinary metabolites over time, (iii) nutrition and (iv) growth (Table 2 and Figure 2). These were used to develop a summary of factors affecting metabolic maturation in infants born preterm (Figure 3), and describe metabolites associated with each of the themes (Table 2).

### 3.4. Category 1: Metabolic|Maturation

Four studies characterised changes to the urinary metabolome in preterm infants associated with postnatal maturation in the first few days [35], and first few weeks of life [34,37,45,46]. They identified postnatal changes to the metabolism of glucogenic amino acids, the tricarboxylic acid (TCA) cycle and choline metabolism. These changes correlate with both post-menstrual age (PMA) and gestational age at birth, demonstrating a unique preterm pattern of metabolic maturation.

### 3.5. Category 2: Metabolic|Signatures

Nine studies [35,36,37,38,39,40,44,45,48] included suggested a distinct metabolic signature of prematurity. Preterm birth was associated with deficiencies in amino acid, carbohydrate, and fatty acid metabolism pathways. Extremely preterm infants had the most significant metabolic aberration with variation in metabolites of tyrosine metabolism including tyrosine, tryptophan, and phenylalanine biosynthesis along with the TCA cycle including arginine and proline metabolism, consistent with a role in foetal maturation. None of the included studies addressed issues relating to early life metabolic instability, hyperglycaemia, and hypertriglyceridemia although Morniroli et al. [48] reported higher losses of glucose in urine of preterm infants compared to those at term.

### 3.6. Category 3: Metabolic|Nutrition

Seven studies [37,40,42,43,44,46,47] examined the effects of differing nutritional sources on the urinary metabolites of preterm infants. Markers of oxidative stress are higher in preterm than term infants. Nutrition can alter these markers of oxidative stress, with parenteral nutrition (PN), as well as formula feeding leading to higher levels being excreted when compared to breast feeding. Urinary metabolites of choline metabolism are increased in response to breast feeding.

### 3.7. Category 4: Metabolic|Growth

Six studies [34,37,40,44,46,48] commented on the metabolic profiles of preterm infants with regard to growth parameters. Only two studies directly compared the urinary metabolomes of preterm infants with differing growth profiles. Hulsemann et al. [34] who found infants with stagnating or decreasing weight to have higher 3-methylhistidine/creatinine ratios. Moltu et al. [37] found no difference in urinary metabolic profiles between preterm infants fed an interventional enhanced nutritional plan and the controls, despite the intervention arm demonstrating significantly better growth.

## 4. Discussion

This scoping review has outlined the current understanding of metabolic maturation and the distinct metabolic profiles associated with prematurity. Metabolic maturation can be defined the relationship to ‘biochemical maturity relative to chronological age’ [13]. Preterm birth was associated with deficiencies in amino acid, carbohydrate, and fatty acid metabolism pathways. This seems to be followed by an increase in glucogenic amino acids TCA cycle metabolites and urinary choline metabolites following birth, which correlate with both premenstrual age (PMA) and gestational age at birth, demonstrating a unique preterm pattern of metabolic maturation. Markers of oxidative stress are higher in preterm than term infants, though these seem modifiable by nutrition, with parenteral nutrition (PN) and formula feeding leading to higher levels being excreted compared to breast feeding.

However, there are several gaps in the current knowledge base, including (i) what is the normal pattern of metabolic maturation for preterm infants, (ii) how metabolic signatures may vary in those infants with metabolic instability (as illustrated by an intolerance of glucose or lipid for example) compared to those who are tolerant of parenteral and enteral nutrition, (iii) what the efficacy of nutritional interventions could be to facilitate metabolic maturation and improve growth outcomes and (iv) whether there is an opportunity to develop reference standards for metabolic maturity, i.e., metabolism may be related to gestational age/corrected gestational age, rather than chronological age.

Briefly, metabolic functions can be split into two categories, bioenergetic functions and metabolic signalling functions. Bioenergetic functions, are highly regulated, supporting canonical metabolic activity such as providing energy or cellular building blocks. Metabolic signalling functions play an instructive or modulatory role in the regulation of metabolic pathways, with metabolites being the rate limiting substrate for epigenetic modification and post-translational modifications [6]. By combining advances in both metabolomic analytics and data analysis with anthropometry it may be possible to define nutritional phenotypes based upon metabolic maturity [6]. With the advent of high throughput analytic techniques to quantify components of biological samples, it increasingly possible to consider the development a more nuanced approach to medical and nutritional management for a whole range of conditions [19].

An elegant study completed by Giallourou et al. [13] demonstrated a potential way metabolites of interest within a paediatric population may be used to assess the efficacy of nutrition interventions. The group characterised changes in urinary metabolic profile of infants (*n* = 1131) from resource constrained settings over 3 continents over time during the first 1000 days of life. Findings suggest that biochemical immaturity during the first two-years-of-life, is associated with poorer growth outcomes, which were evident from as early as three months of age and persisted until the end of the second year of life. Linear and ponderal growth were associated with eight age-dependent metabolic signatures, from which phenome age z score (PAZ) reference curves were developed. The use of PAZ for these metabolites of interest provided the opportunity to determine an infant’s position along this metabolic maturation continuum. In the future, there may be the potential to quantify the effectiveness of a nutrition intervention in real time, as well as targeting the individual infants metabolic age rather than chronological age. This is an attractive model for optimising nutrition support in preterm infants, especially those with metabolic instability, as it provides a non-invasive way to measure nutritional responsiveness in preterm infants together with the opportunity to offer interventions targeted to more precise metabolic predisposition [13].

As growth failure is linked to increased risk of metabolic disease later in life, developing nutrition interventions favouring growth in all children affected by malnutrition is imperative [51], including infants born preterm [13]. Metabolic pathways are also influenced by epigenetic marks early in life [52], and this overlaps with changes in metabolic signatures during the evolution of carbohydrate metabolism which coincide with increasing intestinal uptake of disaccharides in the growing infant. Myoinositol plays an essential role in glucose metabolism and transport, as well as being a precursor for several secondary messaging pathways related to intracellular insulin signalling. Myo-inositol is also a component of structural and signalling lipids such as phyosphatidylinositol [53]. Prematurity also affects metabolic pathways involving hydroxyproline, creatine and myo-inositol [54,55,56,57,58], which may contribute to future cardiometabolic disease [44,54]. This temporal relationship has been eloquently described in a small cohort of pre-pubertal children (4–9 years of age) who were SGA at birth. Myo-inositol (urine) levels were decreased by 4-fold in SGA catch-up growth compared with non-catch-up growth. Transcriptomic analysis identified myo-inositol was associated with gene clusters coding for insulin and insulin like growth factor 1 (IGF-1) children [53].

Preterm infants are known to have altered body composition, which has implications for future cardiometabolic disease risk [59,60] and developing PAZ for metabolites associated with neurodevelopment and body composition (particularly lean mass) may serve as a useful reference against which to identify metabolic age compared to chronological age. Betaine and choline, are important precursors for acetylcholine (a neurotransmitter) and phospholipid (an important structural and signalling component of cell membrane), and low levels in animals are associated with neurodevelopmental delay [61]. Choline is a also a precursor for betaine synthesis which is used to form homocysteine and methionine, essential for protein synthesis and linear growth [62] and higher urinary levels of choline in the first few weeks of life are seen in breastfed infants [63]. A low urinary 3-methylhistidine/creatinine ratio has been shown to be positively correlated with body weight and tissue accretion [63]. Preterm infants with plateauing or decreasing weight have been shown to have 3-methylhistidine/creatinine ratios above normal range [34], and developing PAZ for these metabolites would complement existing work [13,44]. Other significant differences in urinary metabolic signatures in preterm infants include increased 3-hydroxyisovalerate (3-HVA), with decreased dimethylamine (DMA) and 1-methylhistidine which are related to gut microbiome and muscle protein turnover [44]. Aberrance with regard to these metabolites leads to poor nutrient utilisation and development of skeletal and lean muscle mass. Finally, with regard to energy balance, preterm infants have significantly lower urinary concentrations of succinic acid and lactose compared to term infants [44,62] and this in part may be due to age related differences in TCA cycle activity. Higher losses of these end products of metabolism appear to be related to growth faltering resulting in poor weight gain [63], suggesting there may be windows of opportunity for intervention if higher urinary levels than phenome age z scores were found. Further research is required to understand the temporal relationships between urinary metabolites of interest and growth in preterm infants.

## 5. Limitations

This is a scoping review to present the current range of evidence specific to urinary metabolites in preterm infants compared to healthy newborns. A significant issue that this review highlights is the relative lack of longitudinal data describing metabolic maturation within these infant cohorts, which is why the literature included in this scoping review explores what is known about urinary metabolomics in preterm infants. Given this, it was not possible to meta-analyse the results or reliably identify metabolites associated with metabolic stability and growth to allow the development of phenome age z scores.

## 6. Future Research Priorities

Future research is required to describe and define the normal range for urinary metabolites in healthy infants and those with complex disease and of different gestational age to allow the development of PAZ charts for metabolites of interest. This in turn may allow age, and disease specific nutritional interventions. As suggested by Gallouri et al. [13] a priority should be to develop age-specific reference curves for urinary metabolites in preterm infants compared to healthy infants. However, the development of aggregated PAZ for metabolites of interest requires large numbers and longitudinal data. Collaborative efforts to develop these would provide a unique opportunity to further our insight into better supporting ideal growth within these vulnerable infant cohorts.

Developing a better understanding of this relationship [6,13] will help (i) refine our understanding of phenotypic and metabolic responses to nutritional interventions, (ii) provide an opportunity to identify nutritional supplementation, (iii) define age related reference ranges for specific metabolites and (iv) identify specific windows in which targeted supplementation might improve growth outcomes considering metabolic maturity rather than chronological maturity [13,24,25,26].

## 7. Conclusions

The results of this scoping suggest that preterm birth is associated with particular metabolic signatures, and that these signatures change in relation to both increasing PMA and in response to certain patterns of nutrition. However, considerable gaps in our knowledge remain, relating to metabolic maturation of infants, especially those born preterm. Although medical and nutritional management of these infants has significantly improved, a proportion continues to be growth constrained despite adequate nutritional support, for reasons that are unclear. Characterising metabolites of interest and developing PAZ for metabolites associated with the metabolic maturation and growth may elucidate windows of opportunity for nutrition supplementation allowing early intervention before growth failure is identified using anthropometry alone.

## Figures and Tables

**Table 1:** Studies describing urinary metabolites in preterm infants [34,35,36,37,38,39,40,41,42,43,44,45,46,47,48].

Title	Author, Year, Published	Methodology	Study Numbers	Patient Characteristics & Inclusion Criteria	Exclusion Criteria	Study Aim/Method	Main Findings	Over Arching Theme
3-Methylhistidine/creatinine ratio in urine from low-birth-weight infants: Statistical analysis	Hulsemann et al., 1988 [34], Annals of Nutrition & Metabolism	Observational cohort, single centre	30 (23 preterm + 7 term SGA)	Preterm infants (GA 30–36 weeks, age: 9–83 days postpartum) Term SGA infants (2–30 days postpartum)	Infants with major clinical problems (undefined), any infant cared for in the intensive care ward	To assess if the urinary 3-methylhistidine/creatinine ratio is constant over 24 h, as well a statistical analysis of the observed variability in this ratio among different children and in the same child on different days.	1. Diurnal variation of the 3-methylhistidine/creatinine ratio is negligible in any given individual 2. Variability is found as a function of the day of sampling-hypothesised to be due to the corresponding current metabolic state of the individual 3. Infants with stagnating or decreasing weight had higher 3-methylhistidine/creatinine ratios and so this can potentially be used to assess current metabolic state in low-birth-weight infants.	4
Metabolic changes in early neonatal life: NMR analysis of the neonatal metabolic profile to monitor postnatal metabolic adaptations	Georgakopoulou et al., 2020 [35], Metabolomics	Observational cohort, 2 centre	153 (141 term 12 late preterm)	Term infants (GA 37–40 weeks) Late preterm (GA 35–37 weeks)	Nil specifically mentioned	H NMR spectroscopy was used to compare the metabolic urinary profiles from the first and third days of life, assessing the impact of; delivery mode, prematurity, maternal smoking, gender, nutrition, and neonatal jaundice.	1. From day 1 to day 3 multiple changes are noted in the urinary metabolic profiles of healthy term infants. Specifically stronger signals of creatinine, taurine, myo-inositol and weaker signals of creatine and glycine are seen on the first day of life when compared to the third day of life. 2. Trends in differentiation of metabolite levels between late preterm and term infants and observed at day 1 but lost by day 3. 3. There are specific differences between the urinary metabolic profiles of male and female infants, as well of those whose mothers who smoked during pregnancy.	1, 2
H^1^ NMR-based metabolomic analysis of urine from preterm and term neonates	Atzori et al. [36]. 2011 Frontiers in Bioscience	Observational cohort, 2 centre	67 (26 term + 41 preterm infants)	Term infants (GA 37–40 weeks) Preterm infants (GA < 37 weeks)	Nil specifically mentioned	H^1^ NMR spectroscopy was used to analyse the urinary metabolic profiles of term and preterm infants, from samples collected within the first 12 h of life, to identify any gestational age-related differences.	1. The urinary H^1^ NMR profile of premature neonates is different to that of full-term neonates. 2. Profiles also vary between different groups of preterm infants. (Those born at 23–32 weeks compared to those of 33–36 weeks GA). 3. Individual metabolites discriminating between the groups were: Hippurate, tryptophan, phenylalanine, malate, tyrosine, hydroxybutyrate, *N*-acetyl-glutamate, and proline. It is therefore suggested that amino acid biosynthesis and metabolism are the key metabolic mechanisms underlying foetal and perinatal maturation processes.	2
Urinary metabolite profiles in premature infants show early postnatal metabolic adaptation and maturation.	Moltu et al. [37]. 2014 Nutrients	Randomised control Trial, 2 centre	50 (24 intervention, 26 control)	Preterm infants with birth weight (BW) < 1500 g: Intervention: GA 28.1 weeks (25.0–33.6) BW 940 g (460–1311) Control: GA 28.5 weeks (24.0–32.6) BW 1083 g (571–1414) (mean, range)	Congenital malformations, chromosomal abnormalities, critical illnesses with short life expectancy, clinical syndromes known to affect growth and development	To use H NMR spectroscopy to assess the urinary metabolic profile of premature infants randomised to either a standard or an enhanced diet.	1. Enhanced nutrition did not appear to affect the urinary metabolic profiles greater than individual variation. 2. Infants given enhanced nutrition show greater growth velocity, but no changes in their urinary metabolic profile. 3. In all infants the glucogenic amino acids glycine, threonine, hydroxyproline and tyrosine, as well as the metabolites of the TCA (succinate, oxoglutarate, fumarate and citrate) increased during the early postnatal period. 4. The metabolite changes correlated with gestational age at birth and chronological age. 5. Threonine and glycine levels were elevated in first-week urine samples of the small for gestational age infants compared to appropriate for gestational age infants. 6.Neither sex nor the presence of infections had a significant effect on metabolic profile.	1, 3, 4
Comprehensive analysis of the l-arginine/l-homoarginine/nitric oxide pathway in preterm neonates: potential roles for homoarginine and asymmetric dimethylarginine in foetal growth	Buck et al. [38]. 2017 Amino Acids	Observational cohort, single centre, healthy preterm infants	106	51 male infants and 55 female infants, GA 23 + 6–36 + 1 weeks, Group 1: *n* = 31 GA 23 + 6–29 + 6 BW 1.025 ± 0.292 kg, Group 2: *n* = 75 GA 30 + 0–36 + 1 BW 1.800 ± 0.288 kg.	Infection, sepsis, intraventricular haemorrhage > 2°, congenital disorders and/or chromosomal aberrations, pulmonary hypertension, mandatory ventilation, and infant respiratory distress syndrome > 3°	To investigate and describe the Arg/hArg/NO pathway in healthy preterm infants.	All enterally fed with formula or a combination of breast/formula milk. 10 had additional PN 1. ADMA (asymmetric dimethylarginine) and hArg (l-homoarginine) plasma levels were significantly higher in the extremely preterm infants than preterm infants’ group. 2. Urinary ADMA, SDMA and hArg did not correlate with GA, weight, or head circumference in either group. 3. There was no difference with respect to Arg in the plasma or nitrite/nitrate in the plasma or urine. 4. hArg seems to be of higher significance for the female than for the male foetus. Therefore, it is proposed that ADMA and hArg are involved in foetal growth, and that this manner is dependent on the gender	2
Metabolic products in urine of preterm infants characterized via gas chromatography-mass spectrometry	Hao et al. [39]. 2015 International Journal of Clinical and Experimental Medicine	Observational case–control, single centre	92 (45 term + 47 preterm)	Term infants: GA 37–41 weeks Preterm infants: GA 28–36 weeks Note: All infants were formula fed from the 3rd postnatal hour	Foetal distress, birth asphyxia, neonatal complications within the first 6 postnatal hours, APGAR score < 8, abnormal blood gas or lactate, requirement for medical treatment. Maternal medical history of chronic or infectious disease, malnutrition, smoking, alcohol, or drug use.	To characterise the metabolic products of urine associated with preterm birth using gas chromatography on samples obtained within the first 24 h of life. Specifically, the levels of urinary lysine, phenylalanine, histidine, ornithine, fumaric acid, malic acid, succinic acid, lactose, stearic acid, and 4-hydro phenylacetic acid in the urine of preterm infants was compared to that of term.	1. Normalized concentrations of all measured metabolites were significantly lower in preterm infants when compared to full-term infants, some were undetectable. (Lysine, phenylalanine, histidine, ornithine, fumaric acid, malic acid, succinic acid, lactose, stearic acid and 4-hydroxyphenylacetic acid). 2. Inferred that Amino acid, carbohydrate and fatty acid metabolism defects exist in preterm infants.	2
Human milk enhances antioxidant defences against hydroxyl radical aggression in preterm infants	Ledo et al. [40]. 2009 American Journal for Clinical Nutrition	Observational case–control, single centre	83 (Human milk *n* = 29 Preterm formula *n* = 34 Term control group *n* = 20)	Human milk: GA 32 weeks (26, 36) Formula: GA 33 weeks (29, 36) (GA median (95% CI) BW: Human milk 1495 ± 497 g Formula fed: 1743 ± 435.1 g	Acute perinatal or chronic postnatal disease: currently on supplemental oxygen, medications, mineral, or vitamin supplementation, blood transfusion in the 2 weeks prior to enrolment, severe congenital abnormality, chromosomal abnormality, required GI surgery, required PN	To determine the effect of human milk on markers of oxidative stress.	1. Preterm: GA < 37 weeks, Full enteral feeding either exclusively with human milk (own mother’s milk or donor) or with preterm formula, consistent and adequate weight gain the week before enrolment; Controls: healthy, term, fed human milk 2. Both preterm groups, when compared with term new-borns, had significantly higher urinary markers of oxidative stress. 3. The formula fed preterm infants eliminated significantly higher amounts of 8-oxodG and o-Tyr than the preterm infants fed human milk, leading to the conclusion that prematurity is associated with protracted oxidative stress, from which human milk is partially protective 4. When all the data was combined there was a significant correlation between markers of oxidative stress and birth weight.	2, 3, 4
Comparison between tryptophan methoxy indole and kynurenine metabolic pathways in normal and preterm neonates and in neonates with acute foetal distress	Munoz-Hoyas [41] 1998 European Journal of Endocrinology	Observational cohort, single centre	Total 112: 42 control, 30 preterm, 40 foetal distress	Preterm: < 37 weeks GA, Term infants suffering from foetal distress, Healthy term controls Mothers had one or more of; high risk pregnancy, obstetric antecedents or pregnancy diseases	Neurological or endocrine pathology	To analyse the kynurenine and methoxy indole metabolic pathways of tryptophan to identify changes in premature neonates and in neonates suffering from foetal distress.	1. Diurnal differences exist cord in blood melatonin concentration and the urinary excretion of kynurenine- with greater concentrations of Kynurenine in the day and greater concentrations of Melatonin at night. This diurnal pattern is blunted in preterm infants and those with foetal distress.	2
Choline-related metabolites influenced by feeding patterns in preterm and term infants	Shoji et al. [42]. 2018 The Journal of Maternal-Foetal & Neonatal Medicine	Observational cohort, single centre	39 (13 term breast fed, 6 term formula fed, 11 preterm breastfed, 9 preterm mixed feeding)	Term Breast: GA 39.2 ± 1.2 weeks BW 2962.8 ± 296.9 g Term formula: GA 38.1 ± 0.1 weeks, BW 2997.3 ± 181.2 g Preterm Breast: 29.7 ± 1.4 weeks, BW 1139.4 ± 260.1 g Preterm mixed: 30.1 ± 1.0 weeks, BW1223.6 ± 238.2 g (mean ± SD)	Term: Perinatal complications including asphyxia, infection, bleeding, Preterm: Major congenital abnormalities, metabolic disorders, maternal diabetes requiring insulin, chronic hypertension, or intrauterine infection	To examine the choline status of term and preterm infants using analysis of urinary excretion of choline metabolites. (Choline, *N*, *N*-dimethylglycine, Sarcosine, and Betaine)	1. Type of feeding affects choline metabolism 2. Urinary excretion of choline metabolites (Choline, *N*, *N*-dimethylglycine, Sarcosine, and Betaine) was significantly higher in term breast fed infants than term formula fed infants. 3. Urinary excretion of Choline, Betaine, and Sarcosine was not significantly different between the preterm breast fed and term breast fed infants.	3
Suppressive effects of breast milk on oxidative DNA damage in very low birthweight infants	Shoji 2003 et al. [43] Archives of Disease in Childhood Foetal and Neonatal Edition	Observational case control, single centre,	29 (15 breast fed, 14 formula fed)	Breast fed: 8 male 7 female, mean GA 29.2 weeks SD 2.3, mean BW 1231 g, SD 298 Formula fed: 8 male 6 female, mean GA 28.7 weeks SD 2.0, mean BW 1182 g SD 281 Birth weight < 1500 g, cared for in neonatal intensive care unit,	Congenital abnormalities	To examine the antioxidant effects of breast milk in very low birth weight infants	1. 8-OHdG is known to be a marker for in vivo oxidative DNA damage. 2. Urinary 8-OHdG excretion at 14 and 28 days of age is significantly lower than that at 2 and 7 days of age in breast fed infants. 3. Urinary 8-OHdG excretion is also lower at days 14 and 28 in breast fed infants when compared to formula fed infants. 4. In formula fed infants there is no significant difference in urinary 8-OHdG excretion at 2, 7, 14, and 28 days of age. Conclusion: Evidence of the antioxidant effect of human milk in very low birth weight infants.	3
New-born Urinary Metabolic Signatures of Prematurity and Other Disorders: A Case Control Study	Diaz et al. [44]. 2016 Journal of Proteome Research	Observationalcase control, single centre	148: (46 Controls, 102 with specific disorders as listed: (1) late preterm = 17, (2) Respiratory depression = 10, (3) LGA = 18, (4) Congenital malformation = 9 (5) PROM = 33 and (6) Jaundice = 12.)	Healthy Controls + New-borns with specific disorders as follows: Late Preterm infants (GA 33–36 weeks) Infants with respiratory depression following delivery, LGA, congenital malformations, PROM, jaundice	Nil specifically mentioned	To assess, by H NMR spectroscopy, the urinary metabolic signature of prematurity whilst also examining potential confounders and signature specificity by comparing with the metabolic signatures of other disorders.	1. Overall the metabolic signature of prematurity was comprised of changes in 25 identified, and several more unassigned, metabolites. Those identified suggest disturbances in nucleotide metabolism, lung surfactants biosynthesis and renal function, along with enhancement of TCA cycle activity, fatty acids oxidation, and oxidative stress. 2. Gender and mode of delivery impact urinary metabolic profile. 3. Profile changes were also noted for new-borns experiencing respiratory depression, LGA and malformations but these were distinct from the changes of prematurity.	2, 4
Urinary metabolites of oxidative stress and nitric oxide in preterm and term infants	Farkouh et al. [45]. 2006 Biology of the Neonate	Observational cohort, 2 centre study	102 (82 preterm 20 term)	Preterm: GA 27.4 ± 2.6 weeks BW 1048 ± 407 g Term: GA 38.4 ± 1.6 weeks BW 3210 ± 4467 g (mean ± SD)	All: Major congenital abnormality, chromosomal anomaly, received iNO or multivitamin supplementation, Term controls: SGA, requiring medical support	To determine the effects of clinical interventions in preterm infants on markers of oxidative stress and nitric oxide metabolism. The substrate markers measured were levels of urinary peroxides and nitrates/nitrites, respectively.	1. Premature infants had significantly higher urinary peroxide levels than term infants. Urinary nitrite/nitrate levels were not significantly different. 2. Infants receiving PN had significantly higher urinary peroxide levels than those not receiving PN. 3. Administration of Indomethacin resulted in lower nitrate and nitrite levels. 4. Receiving mechanical ventilation or high inhaled Fi02 did not affect either marker.	2, 3
Fatty acid profiles, antioxidant status, and growth of preterm infants fed diets without or with long-chain polyunsaturated fatty acids: a randomized clinical trial	Koletzko et al. [46]. 2003 European Journal of Nutrition	Double blind, randomised control trial	Total: 46 (29 formula fed-15 LCP supplemented formula, 14 low LCP formula, 17 breast fed controls)	Preterm, ‘stable’ clinical condition, BW < 1800 g Breast Fed Controls: GA 31 ± 2 weeks, BW 1440 ± 288 g, Supplemented formula: GA 30 ± 2 weeks, BW 1145 ± 288 g, Unsupplemented formula: GA 30 ± 3 weeks, BW 1177 ± 344 g. (mean ± SD)	Artificial ventilation, need for supplemental oxygen with Fi02 > 0.3, presence of apparent genetic, gastrointestinal, or metabolic disorders	To examine the effect of an infant formula enriched with *n*-6 and *n*-3 long chain polyunsaturated fatty acids on plasma fatty acids, antioxidant studies and growth in preterm infants. Antioxidant status was assessed using urinary malondialdehyde as a marker of oxidative stress.	1. Plasma long chain polyunsaturated fatty acid levels similar to those of breast fed infants can be achieved with a supplemented formula. 2. Urinary malondialdehyde excretion was significantly higher from formula fed infants than infants fed human milk. There was however no difference between the formula fed groups, suggesting there to be no adverse effects of the enriched formula with regard to oxidative stress. 3. No difference in growth was seen between the groups over the study period.	3, 4
Urinary metabolomic profile of preterm infants receiving human milk with either bovine or donkey milk-based fortifiers	Giribaldi et al. [47]. 2020 Nutrients	Single blinded, randomised control trial	54 (Bovine-Human milk = 27, Donkey-Human milk = 27)	GA < 32 weeks and/or BW ≤ 1500 g Bovine-Human milk: BW 1174 g (326), Donkey-Human milk: BW 1227 g (302) (mean g (SD))	Severe gastrointestinal pathology, chromosomal abnormality, major congenital abnormality, metabolic disease, disseminated intravascular coagulopathy, patent ductus arteriosus, renal failure	To analyse the urinary metabolome of infants fed human milk fortified with bovine and donkey milk-based fortifiers. The metabolic profiles were analysed at day 1 and day 21 of the intervention using H NMR spectroscopy.	1. The urinary metabolic profiles of preterm and very low birth weight infants show postnatal adaptation. Changes common to all infants studied included: increasing urinary betaine, citrate, succinate, formate, alpha-ketoglutarate and *N*, *N*-dimethylglycine, and decreasing *N*-acetyl tyrosine. 2. Bovine and donkey milk fortifiers give distinct urinary metabolic profiles, due to the differing nutrient qualities. There was higher excretion of galactose in the donkey milk group but higher carnitine, choline, lysine, and leucine in the bovine group.	1, 3
Is the body composition development in premature infants associated with a distinctive nuclear magnetic resonance metabolomic profiling of urine?	Morniroli et al. [46] 2019 The Journal of Maternal-Fetal & Neonatal Medicine	Observational cohort, single centre	20 (13 preterm, 7 term)	GA ≤ 32 weeks, singleton pregnancy, exclusively formula fed Preterm: BW 1113.4 g (CI 956.8–1270) GA 29.7 (CI 28.6–30.8) Term: BW 3285 g (CI 2907–3663) GA 38.7 (CI 37.9–39.5)	Congenital malformations, chromosomal abnormalities, chronic lung disease, necrotizing enterocolitis, Papillae grade intraventricular haemorrhage > 2 or any renal, endocrine, or cardiac congenital disease	To compare the metabolomic profile of preterm infants at term and at 3 months with that of term infants, and to determine if there is any association with body composition development.	1. At term-corrected age, fat mass, both in terms of percentage and absolute content, was significantly higher in preterm infants than in full-term infants. At 3 months corrected the body composition parameters were similar between the two groups. 2. There were significant differences in the urinary metabolic profiles of the two groups. At term corrected the preterm group exhibited higher urinary citrate, choline/phosphocholine, lactate, betaine, and glucose but lower myo-inositol, creatinine, dimethylamine, and ethanolamine. At 3 months corrected the preterm group exhibited higher urinary creatinine, choline/phosphocholine and lactate and a lower betaine, glycine, and citrate.	1, 2, 4

Abbreviations: 8-OHdG = 8-hydroxydeoxyguanosine; ADMA = asymmetric dimethylarginine, Arg = l-arginine, BW = birth weight, CI = confidence interval, Fi02 = fractional inspired oxygen, HArg = l-homoarginine; H^1^ NMR = Hydrogen nuclear magnetic resonance spectroscopy iNO = inspired nitric oxide, GA = gestational age, GI = gastrointestinal, LGA = large for gestational age; NO = nitric oxide, PN = parenteral nutrition, PROM = prolonged rupture of membranes, SGA = small for gestational age, SADMA = symmetric dimethylarginine TCA = tricarboxylic acid.

**Table 2:** Content analysis of metabolite maturation in infants born preterm.

Theme	Comparison	Key Findings	Associated Metabolic Pathways
**1. Metabolic Maturation**	Preterm infants’ maturation over time	Increasing glucogenic amino acids; glycine, threonine, hydroxyproline and tyrosine Increasing metabolites of the tricarboxylic acid cycle (TCA); succinate, oxoglutarate, fumarate, alpha-ketoglutarate, citrate Increasing urinary choline metabolites; betaine, *N*,*N*-dimethylglycine	Succinate, oxoglutarate, fumarate, alpha-ketoglutarate, citrate are all part of the TCA cycle, (also known as Krebs or citric acid cycle). The TCA cycle is the main source of energy for cells. The TCA cycle is part of the larger glucose metabolism whereby glucose is oxidized to form pyruvate, which is then oxidized and enters the TCA cycle as acetyl-CoA. Gluconeogenic amino acids also enter the TCA cycle [37]. Choline is involved in several pathways associated with neurotransmitters and is important component of brain development and neurocognition. Choline is also oxidized in the mitochondria to betaine. The methyl groups of betaine are used to re-synthesize methionine from homocysteine, providing methionine for protein synthesis and transmethylation reactions [42].
	Term and later preterm infants from day 1 to day 3 of life	Increasing creatine, glycine, betaine, alanine, galactose, formate, dimethylglycine, lysine and ethanolamine Lower taurine, myo-inositol, trigonelline, creatinine, hypoxanthine, *N*-methylnicotinamide, cis-aconitate, ascorbate and lactose.	Alanine, lysine and creatine are associated with amino acid and nitrogen metabolism. Biosynthesis of creatinine is associated with increase muscle mass in infants [44]. The inositol pathway is formed of eight inositol isomers, all of which are formed from the epimerisation of myo-inositol. Myo-inositol is involved in the intracellular transmission of insulin’s metabolic signal and is also important for the oxidative use of glucose and its storage as glycogen. Aberrance in myo-inositol is associated with insulin resistance [49].
**2. Metabolic Signatures**	Preterm infants’ vs. Term Infants	**During first 24 h of life:** Higher *N*-methyl-nicotinamide Lower ethanolamine **During Neonatal Period:** Lower essential amino acids; lysine, phenylalanine, histidine, Lower amino acid metabolites; ornithine, methyl-histidine Lower carbohydrate metabolites; lactose Lower fatty acid metabolites; stearic acid, 4-hydroxyphenylacetic acid Lower ketone bodies; acetone and 3-hydroxybutyrate Changes to TCA cycle intermediates; fumarate, malate, succinate, citrate Higher myo-inositol Higher 3-hydroxyisovalerate—A marker of reduced biotin status Higher markers of oxidative stress: urinary peroxide, oxidative bases of DNA and oxidative derivatives of Phenylalanine Blunted diurnal variation of tryptophan methoxyindole and kynurenine metabolic pathways in preterm infants Higher levels of L-homoarginine, asymmetric dimethyl-arginine (ADMA) and symmetric dimethylarginine (SDMA) in preterm infants >30 weeks gestational age vs. <30 weeks gestational age Inconsistent results reported fumarate, succinate and lysine-reported to be lower by Hao et al. [39] but higher by Diaz et al. [44] **Term corrected:** Higher urinary citrate, choline/phosphocholine, lactate, betaine and glucose in pre-term infants Lower myo-inositol, creatinine, dimethylamine and ethanolamine in pre-term infants **Three months corrected:** Higher urinary creatinine, choline/phosphocholine and lactate in preterm infants Lower betaine, glycine and citrate in preterm infants	TCA cycle metabolites fumarate, succinate, lysine Hao et al. [39] report lower urinary levels, but Diaz et al. [44] report higher levels. Gluconeogenic amino acids glycine plays an important role in metabolic regulation, anti-oxidative reactions, and neurological function [50]. Metabolites associated with metabolism of carbohydrates, fatty acids and amino acids [39]. Lysine is the primary limiting amino acid for protein synthesis and has a significant role in calcium absorption, muscle mass accretion, alleviation of pain and inflammation [39]. Phenylalanine is a building block for protein and histidine is an essential amino acid in infants up to 6 months of age, inadequate consumption results in growth failure and increased loss of nitrogen [39]. Ornithine, fumaric acid and malic acid have important roles in amino acid metabolism and energy conservation [39]. Lower levels of amino acids in urine of preterm infants may be due to lower protein deposition of essential amino acids or lack of metabolic enzymes required for nutrient utilization [39]. Lower lactose levels may occur due to lower lactase enzyme activity in preterm infants intestinal tract with lower sugar metabolism and storage [39]. Lower ketone bodies and ketogenic amino acid lysine may be associated with deranged energy metabolism, as well as an increased reliance of fatty acids as a source of energy, with lower levels of lactate and potential enhanced use of pyruvate in the TCA cycle Lower metabolites related to the gut microbiome; dimethylamine (DMA) and 1-methyl-histidine and reduced biotin status [44]. ADMA and l-homoarginine are part of the nitric oxide pathway which is associated with many physiological processes including regulation of blood pressure, inhibition of platelet aggregation and neurotransmission [38]. Tryptophan metabolism in the brain involves the methoxyindole and kynurenine metabolic pathways, includes the metabolite melatonin, associated with circadian rhythm with tryptophan degradation occurring via methyoxyindoles [41]. The most important metabolic cycles related to variations in metabolites between preterm and term infants were; tyrosine metabolism (tyrosine and tryptophan); phenylalanine biosynthesis; TCA cycle; arginine and proline metabolism [36].
**3. Nutrition**	Formula vs. Human milk	8-oxodG, 8-OHdG, o-Tyr and urinary malondialdehyde (markers of oxidative stress) all higher in formula milk groups Choline metabolites higher in breast fed groups	8-oxodG may be used as a measure of oxidative stress and oxidative damage [40,42]. Choline metabolism is associated with lung surfactant synthesis [42,43,44] Urinary peroxides may be used as a measure of oxidative stress [43]
	Parenteral nutrition (PN) vs. Enteral feeding	Higher urinary peroxides in PN group	
**4. Growth**	Birth weight	Markers of oxidative stress inversely correlate with birth weight	Variation in ethanolamine and myo-inositol may also reflect changes in membrane synthesis of phosphatidylinositol (PI) which act as lung surfactants, as such decrease myo-inositol may reflect a temporary increase in PI requirements [40]. 3-methylhistidine is a constituent of actin and myosin of white muscle fibres. It is not reutilised for protein synthesis and can be used as a measure of muscle protein turnover. Creatinine is formed from creatine and creatine phosphatase in muscle and can be used as an indirect measure of lean muscle mass. Urinary 3-methylhistidine/creatinine ratio can be used as an indicator of nutritional and metabolic status. Infants with a higher ratio were more likely to have growth failure [34]. Increased urinary excretion of choline, a betaine precursor, could reflect a potential altered metabolism in preterm infants [48].
	Preterm vs. term infants	At term corrected preterm infants have higher fat mass, urinary citrate, choline/phosphocholine, lactate, betaine and glucose but lower myo-inositol, creatinine, dimethylamine and ethanolamine	
	Preterm infants SGA	Increased threonine and glycine levels in first week of life	
	Preterm infants with stagnating or decreasing weight	Higher urinary 3-methylhistidine/creatinine ratios	
	Preterm infants vs. term infants	Lower fat mass in preterm infants with increase in urinary choline/phosphocholine, betaine and glucose in preterm infants compared to term.	

## Appendix A

**Table A1:** Preferred Reporting Items for Systematic reviews and Meta-Analyses extension for Scoping Reviews (PRISMA-ScR) Checklist.

Section	Item	PRISMA-ScR Checklist Item	Reported on Page
Title			
Title	1	Identify the report as a scoping review.	1
**Abstract**			
Structured summary	2	Provide a structured summary that includes (as applicable): background, objectives, eligibility criteria, sources of evidence, charting methods, results, and conclusions that relate to the review questions and objectives.	1
**Introduction**			
Rationale	3	Describe the rationale for the review in the context of what is already known. Explain why the review questions/objectives lend themselves to a scoping review approach.	3
Objectives	4	Provide an explicit statement of the questions and objectives being addressed with reference to their key elements (e.g., population or participants, concepts, and context) or other relevant key elements used to conceptualize the review questions and/or objectives.	3
**Methods**			
Protocol and registration	5	Indicate whether a review protocol exists; state if and where it can be accessed (e.g., a Web address); and if available, provide registration information, including the registration number.	4
Eligibility criteria	6	Specify characteristics of the sources of evidence used as eligibility criteria (e.g., years considered, language, and publication status), and provide a rationale.	4
Information sources *	7	Describe all information sources in the search (e.g., databases with dates of coverage and contact with authors to identify additional sources), as well as the date the most recent search was executed.	4
Search	8	Present the full electronic search strategy for at least 1 database, including any limits used, such that it could be repeated.	4
Selection of sources of evidence †	9	State the process for selecting sources of evidence (i.e., screening and eligibility) included in the scoping review.	4
Data charting process ‡	10	Describe the methods of charting data from the included sources of evidence (e.g., calibrated forms or forms that have been tested by the team before their use, and whether data charting was done independently or in duplicate) and any processes for obtaining and confirming data from investigators.	4
Data items	11	List and define all variables for which data were sought and any assumptions and simplifications made.	4
Critical appraisal of individual sources of evidence §	12	If done, provide a rationale for conducting a critical appraisal of included sources of evidence; describe the methods used and how this information was used in any data synthesis (if appropriate).	4
Synthesis of results	13	Describe the methods of handling and summarizing the data that were charted.	4
**Results**			
Selection of sources of evidence	14	Give numbers of sources of evidence screened, assessed for eligibility, and included in the review, with reasons for exclusions at each stage, ideally using a flow diagram.	6
Characteristics of sources of evidence	15	For each source of evidence, present characteristics for which data were charted and provide the citations.	7
Critical appraisal within sources of evidence	16	If done, present data on critical appraisal of included sources of evidence (see item 12).	7
Results of individual sources of evidence	17	For each included source of evidence, present the relevant data that were charted that relate to the review questions and objectives.	7
Synthesis of results	18	Summarize and/or present the charting results as they relate to the review questions and objectives.	7
**Discussion**			
Summary of evidence	19	Summarize the main results (including an overview of concepts, themes, and types of evidence available), link to the review questions and objectives, and consider the relevance to key groups.	26
Limitations	20	Discuss the limitations of the scoping review process.	30
Conclusions	21	Provide a general interpretation of the results with respect to the review questions and objectives, as well as potential implications and/or next steps.	31
**Funding**			
Funding	22	Describe sources of funding for the included sources of evidence, as well as sources of funding for the scoping review. Describe the role of the funders of the scoping review.	31

JBI = Joanna Briggs Institute; PRISMA-ScR = Preferred Reporting Items for Systematic reviews and Meta-Analyses extension for Scoping Reviews. * Where sources of evidence are compiled from, such as bibliographic databases, social media platforms, and Web sites. † A more inclusive/heterogeneous term used to account for the different types of evidence or data sources (e.g., quantitative and/or qualitative research, expert opinion, and policy documents) that may be eligible in a scoping review as opposed to only studies. This is not to be confused with information sources. ‡ The frameworks by Arksey and O’Malley (6) and Levac and colleagues (7) and the JBI guidance (4, 5) refer to the process of data extraction in a scoping review as data charting. § The process of systematically examining research evidence to assess its validity, results, and relevance before using it to inform a decision. This term is used for items 12 and 19 instead of “risk of bias” (which is more applicable to systematic reviews of interventions) to include and acknowledge the various sources of evidence that may be used in a scoping review (e.g., quantitative and/or qualitative research, expert opinion, and policy document).

## Appendix B

**Table A2:** Search strategy for PuBMed and study inclusion criteria to do.

Study Selection Criteria (PICOTS)		
	Inclusion Criteria	Exclusion Criteria
**Population**	Preterm infants < 37 weeks gestational age	Infants > 37 weeks gestation age; Exclusion criteria included studies not published in English, infants with other primary pathologies (including metabolic, gastrointestinal, nephrological, neurological or urological) and metabolites described in fluids other than urine
**Intervention**	Mass spectroscopy urinary metabolomics	Mass spectroscopy metabolomics from other biological samples
**Comparison**	Term neonates	Infants with other primary pathologies (including metabolic, gastrointestinal, nephrological, neurological or urological)
**Outcome**	Growth	Growth not reported
**Timing**	Infants < 37 weeks gestational age	Infants > 37 weeks gestational age, or those with other primary pathology
**Setting**	Hospital	Community
**Search Strategy**		
**Search words**	Infants or neonates; Preterm or premature or prematurity; Urinary metabolomics or urinary metabolites; Growth or weight gain; Metabolic maturation or metabolic maturity	
**Limits**	English, human	
**Year range**	Up to February 2022	
**Search example: PUBMED**	((Infants or Neonates) AND (Urinary metabolomics or urinary metabolites)) AND (weight or growth)	
**Expanded search terms**	(“infant, premature”[MeSH Terms] OR (“infant”[All Fields] AND “premature”[All Fields]) OR “premature infant”[All Fields] OR (“preterm”[All Fields] AND “infants”[All Fields]) OR “preterm infants”[All Fields] OR (“infant, premature”[MeSH Terms] OR (“infant”[All Fields] AND “premature”[All Fields]) OR “premature infant”[All Fields] OR (“premature”[All Fields] AND “infants”[All Fields]) OR “premature infants”[All Fields])) AND (((“urinary tract”[MeSH Terms] OR (“urinary”[All Fields] AND “tract”[All Fields]) OR “urinary tract”[All Fields] OR “urinary”[All Fields]) AND (“metabolome”[MeSH Terms] OR “metabolome”[All Fields] OR “metabolomes”[All Fields] OR “metabolomics”[MeSH Terms] OR “metabolomics”[All Fields] OR “metabolomic”[All Fields])) OR ((“urinary tract”[MeSH Terms] OR (“urinary”[All Fields] AND “tract”[All Fields]) OR “urinary tract”[All Fields] OR “urinary”[All Fields]) AND (“metabolite”[All Fields] OR “metabolite s”[All Fields] OR “metabolites”[All Fields]))) AND (“growth and development”[MeSH Subheading] OR (“growth”[All Fields] AND “development”[All Fields]) OR “growth and development”[All Fields] OR “growth”[All Fields] OR “growth”[MeSH Terms] OR “growths”[All Fields] OR (“weight gain”[MeSH Terms] OR (“weight”[All Fields] AND “gain”[All Fields]) OR “weight gain”[All Fields])) AND (((“metabolic”[All Fields] OR “metabolical”[All Fields] OR “metabolically”[All Fields] OR “metabolics”[All Fields] OR “metabolism”[MeSH Terms] OR “metabolism”[All Fields] OR “metabolisms”[All Fields] OR “metabolism”[MeSH Subheading] OR “metabolic networks and pathways”[MeSH Terms] OR (“metabolic”[All Fields] AND “networks”[All Fields] AND “pathways”[All Fields]) OR “metabolic networks and pathways”[All Fields] OR “metabolities”[All Fields] OR “metabolization”[All Fields] OR “metabolize”[All Fields] OR “metabolized”[All Fields] OR “metabolizer”[All Fields] OR “metabolizers”[All Fields] OR “metabolizes”[All Fields] OR “metabolizing”[All Fields]) AND (“maturate”[All Fields] OR “maturated”[All Fields] OR “maturating”[All Fields] OR “maturation”[All Fields] OR “maturational”[All Fields] OR “maturations”[All Fields] OR “maturative”[All Fields] OR “mature”[All Fields] OR “matured”[All Fields] OR “maturer”[All Fields] OR “maturers”[All Fields] OR “matures”[All Fields] OR “maturing”[All Fields] OR “maturities”[All Fields] OR “maturity”[All Fields])) OR ((“metabolic”[All Fields] OR “metabolical”[All Fields] OR “metabolically”[All Fields] OR “metabolics”[All Fields] OR “metabolism”[MeSH Terms] OR “metabolism”[All Fields] OR “metabolisms”[All Fields] OR “metabolism”[MeSH Subheading] OR “metabolic networks and pathways”[MeSH Terms] OR (“metabolic”[All Fields] AND “networks”[All Fields] AND “pathways”[All Fields]) OR “metabolic networks and pathways”[All Fields] OR “metabolities”[All Fields] OR “metabolization”[All Fields] OR “metabolize”[All Fields] OR “metabolized”[All Fields] OR “metabolizer”[All Fields] OR “metabolizers”[All Fields] OR “metabolizes”[All Fields] OR “metabolizing”[All Fields]) AND (“maturate”[All Fields] OR “maturated”[All Fields] OR “maturating”[All Fields] OR “maturation”[All Fields] OR “maturational”[All Fields] OR “maturations”[All Fields] OR “maturative”[All Fields] OR “mature”[All Fields] OR “matured”[All Fields] OR “maturer”[All Fields] OR “maturers”[All Fields] OR “matures”[All Fields] OR “maturing”[All Fields] OR “maturities”[All Fields] OR “maturity”[All Fields])))Translations preterm infants: “infant, premature”[MeSH Terms] OR (“infant”[All Fields] AND “premature”[All Fields]) OR “premature infant”[All Fields] OR (“preterm”[All Fields] AND “infants”[All Fields]) OR “preterm infants”[All Fields]premature infants: “infant, premature”[MeSH Terms] OR (“infant”[All Fields] AND “premature”[All Fields]) OR “premature infant”[All Fields] OR (“premature”[All Fields] AND “infants”[All Fields]) OR “premature infants”[All Fields]Urinary: “urinary tract”[MeSH Terms] OR (“urinary”[All Fields] AND “tract”[All Fields]) OR “urinary tract”[All Fields] OR “urinary”[All Fields]metabolomics: “metabolome”[MeSH Terms] OR “metabolome”[All Fields] OR “metabolomes”[All Fields] OR “metabolomics”[MeSH Terms] OR “metabolomics”[All Fields] OR “metabolomic”[All Fields]urinary: “urinary tract”[MeSH Terms] OR (“urinary”[All Fields] AND “tract”[All Fields]) OR “urinary tract”[All Fields] OR “urinary”[All Fields]metabolites: “metabolite”[All Fields] OR “metabolite’s”[All Fields] OR “metabolites”[All Fields]Growth: “growth and development”[Subheading] OR (“growth”[All Fields] AND “development”[All Fields]) OR “growth and development”[All Fields] OR “growth”[All Fields] OR “growth”[MeSH Terms] OR “growths”[All Fields]weight gain: “weight gain”[MeSH Terms] OR (“weight”[All Fields] AND “gain”[All Fields]) OR “weight gain”[All Fields]Metabolic: “metabolic”[All Fields] OR “metabolical”[All Fields] OR “metabolically”[All Fields] OR “metabolics”[All Fields] OR “metabolism”[MeSH Terms] OR “metabolism”[All Fields] OR “metabolisms”[All Fields] OR “metabolism”[Subheading] OR “metabolic networks and pathways”[MeSH Terms] OR (“metabolic”[All Fields] AND “networks”[All Fields] AND “pathways”[All Fields]) OR “metabolic networks and pathways”[All Fields] OR “metabolities”[All Fields] OR “metabolization”[All Fields] OR “metabolize”[All Fields] OR “metabolized”[All Fields] OR “metabolizer”[All Fields] OR “metabolizers”[All Fields] OR “metabolizes”[All Fields] OR “metabolizing”[All Fields] maturation: “maturate”[All Fields] OR “maturated”[All Fields] OR “maturating”[All Fields] OR “maturation”[All Fields] OR “maturational”[All Fields] OR “maturations”[All Fields] OR “maturative”[All Fields] OR “mature”[All Fields] OR “matured”[All Fields] OR “maturer”[All Fields] OR “maturers”[All Fields] OR “matures”[All Fields] OR “maturing”[All Fields] OR “maturities”[All Fields] OR “maturity”[All Fields]metabolic: “metabolic”[All Fields] OR “metabolical”[All Fields] OR “metabolically”[All Fields] OR “metabolics”[All Fields] OR “metabolism”[MeSH Terms] OR “metabolism”[All Fields] OR “metabolisms”[All Fields] OR “metabolism”[Subheading] OR “metabolic networks and pathways”[MeSH Terms] OR (“metabolic”[All Fields] AND “networks”[All Fields] AND “pathways”[All Fields]) OR “metabolic networks and pathways”[All Fields] OR “metabolities”[All Fields] OR “metabolization”[All Fields] OR “metabolize”[All Fields] OR “metabolized”[All Fields] OR “metabolizer”[All Fields] OR “metabolizers”[All Fields] OR “metabolizes”[All Fields] OR “metabolizing”[All Fields] maturity: “maturate”[All Fields] OR “maturated”[All Fields] OR “maturating”[All Fields] OR “maturation”[All Fields] OR “maturational”[All Fields] OR “maturations”[All Fields] OR “maturative”[All Fields] OR “mature”[All Fields] OR “matured”[All Fields] OR “maturer”[All Fields] OR “maturers”[All Fields] OR “matures”[All Fields] OR “maturing”[All Fields] OR “maturities”[All Fields] OR “maturity”[All Fields]	
